# EGF signaling promotes the lineage conversion of astrocytes into oligodendrocytes

**DOI:** 10.1186/s10020-022-00478-5

**Published:** 2022-05-04

**Authors:** Xinyu Liu, Conghui Li, Jiao Li, Lesi Xie, Zeng Hong, Kang Zheng, Xiaofeng Zhao, Aifen Yang, Xiaofeng Xu, Huaping Tao, Mengsheng Qiu, Junlin Yang

**Affiliations:** 1 College of Life Sciences, Zhejiang University, Hangzhou, 310058 China; 2 College of Life and Environmental Sciences, Hangzhou Normal University, Hangzhou, 311121 China; 3 Key Laboratory of Organ Development and Regeneration of Zhejiang Province, Hangzhou, 311121 China; 4 Department of Eugenics and Genetics, Maternal and Child Health Care Hospital of Guangxi Zhuang Autonomous Region, Nanning, 530003 China; 5 Affiliated Hangzhou First People’s Hospital, Zhejiang University School of Medicine, Hangzhou, 310006 China

**Keywords:** Astrocyte, Oligodendrocyte, Transdifferentiation, EGF, Erk1/2

## Abstract

**Background:**

The conversion of astrocytes activated by nerve injuries to oligodendrocytes is not only beneficial to axonal remyelination, but also helpful for reversal of glial scar. Recent studies have shown that pathological niche promoted the Sox10-mediated astrocytic transdifferentiation to oligodendrocytes. The extracellular factors underlying the cell fate switching are not known.

**Methods:**

Astrocytes were obtained from mouse spinal cord dissociation culture and purified by differential adherent properties. The lineage conversion of astrocytes into oligodendrocyte lineage cells was carried out by Sox10-expressing virus infection both in vitro and in vivo, meanwhile, epidermal growth factor (EGF) and epidermal growth factor receptor (EGFR) inhibitor Gefitinib were adopted to investigate the function of EGF signaling in this fate transition process. Pharmacological inhibition analyses were performed to examine the pathway connecting the EGF with the expression of oligodendrogenic genes and cell fate transdifferentiation.

**Results:**

EGF treatment facilitated the Sox10-induced transformation of astrocytes to O4^+^ induced oligodendrocyte precursor cells (iOPCs) in vitro. The transdifferentiation of astrocytes to iOPCs went through two distinct but interconnected processes: (1) dedifferentiation of astrocytes to astrocyte precursor cells (APCs); (2) transformation of APCs to iOPCs, EGF signaling was involved in both processes. And EGF triggered astrocytes to express oligodendrogenic genes Olig1 and Olig2 by activating extracellular signal-regulated kinase 1 and 2 (Erk1/2) pathway. In addition, we discovered that EGF can enhance astrocyte transdifferentiation in injured spinal cord tissues.

**Conclusions:**

These findings provide strong evidence that EGF facilitates the transdifferentiation of astrocytes to oligodendrocytes, and suggest that targeting the EGF-EGFR-Erk1/2 signaling axis may represent a novel therapeutic strategy for myelin repair in injured central nervous system (CNS) tissues.

**Supplementary Information:**

The online version contains supplementary material available at 10.1186/s10020-022-00478-5.

## Background

The central nervous system (CNS) is mainly composed of neurons, oligodendrocytes (OLs), astrocytes and microglia (Allen and Lyons 2018). While CNS injury leads to apoptosis of neurons and oligodendrocytes in the injured areas, astrocytes in the lesions are activated and become proliferative again. Reactive astrocytes contribute to the formation of glial scars which hinder the regeneration of axons and myelin during regeneration process (Sofroniew and Vinters 2010; Robel et al. 2011). Thus, an ideal strategy for injury repair is to convert these reactive astrocytes to physiologically functional neurons or oligodendrocytes, as it is not only beneficial to the repair of neural circuit and remyelination, but also helpful in reducing glial hyperplasia and reversing glial scars to normal tissues. Somatic reprogramming has opened up a new avenue for tissue repair, and many studies have succeeded in direct conversion between different cell types (Pang et al. 2011; Yang et al. 2013; Najm et al. 2013; Li et al. 2015; Treutlein et al. 2016; Pesaresi et al. 2019).

It has been recently demonstrated that astrocytes can be transdifferentiated to oligodendrocytes, but which appears to be inefficient (Khanghahi et al. 2018; Zare et al. 2018; Ghasemi-Kasman et al. 2018; Farhangi et al. 2019). And it is striking that astrocytes seem to be more prone to convert into oligodendrocytes in pathological tissues (Khanghahi et al. 2018), suggesting some extracellular signals that may exist in the pathological microenvironment are needed to achieve the cell fate switching. Exploring such extracellular factors will not only help to reveal the molecular mechanism underlying the astrocyte-to-oligodendrocyte fate change, but also aid to identify therapeutic targets for improving the efficiency of lineage conversion.

Epidermal growth factor (EGF) is an extracellular ligand that binds to EGFR on the target cell membrane and activates several signaling cascades, converting extracellular cues into appropriate cellular responses. The EGFR, also known as erbB1, is a transmembrane glycoprotein belonging to the erbB family of receptor tyrosine kinases (Galvez-Contreras et al. 2013). After ligand binding, EGFR dimerizes with itself or with its homologs erbB-2, erbB-3, or erbB-4 and autophosphorylates on key cytoplasmic residues. Activated EGFR recruits adapter proteins which in turn activates complex downstream signaling cascades (Normanno et al. 2006). Sox10 belongs to the SRY (sex-determining region Y) box-containing (SOX) gene family (Pingault et al. 2022), which not only plays an important role in oligodendrocyte development (Finzsch et al. 2008), but also has the capability to transdifferentiate other types of cells into oligodendrocyte lineage cells (Najm et al. 2013; Yang et al. 2013; Khanghahi et al. 2018). In this study, we demonstrate that EGF facilitates Sox10 induction of astrocyte transdifferentiation to oligodendrocyte lineage cells both in vitro and in vivo. This effect is manifested by the upregulation of Olig1 and Olig2, and mediated by activation of mitogen-activated protein kinase (MAPK) pathway in an extracellular signal-regulated kinase 1 and 2 (Erk1/2)-dependent fashion.

## Materials and methods

### Preparation and purification of mouse spinal cord astrocytes

Mouse spinal cords were isolated from postnatal day 1 mouse pups, all experimental procedures were carried out in accordance with institutional guidelines for the care and use of laboratory animals, and the protocol was approved by the Animal Ethics Committee of Hangzhou Normal University, China. Spinal cord tissues were diced into ~ 1 mm^3^ pieces, then digested with 0.05% Trypsin/EDTA (w/v, Gibco, Carlsbad, USA) in a 37 °C incubator for 5 min, followed by adding D/F20S medium (DMEM/F-12 supplemented with 20% v/v FBS, all from Gibco, Carlsbad, USA) to terminate digestion. Tissues were pipetted up and down until homogenized, and then transferred into a 15 ml centrifuge tube and centrifuged at 200×*g* for 5 min at room temperature. After removing the supernatant, D/F20S medium was added to resuspend the pellet, the suspension was transferred into flasks (Corning, NY, USA) and incubated with 5% CO_2_ at 37 °C for 72 h. Culture medium was replaced every other day. When mixed glial cultures became confluent, and floating cells were removed by gently rinsing twice with 1× PBS, before fresh D/F20S medium was added. Culture flasks were placed in incubator to equilibrate the medium for 2 h, followed by shaking for 15–18 h (37 °C, 250 rpm) with tightened caps on an orbital shaker model 420, Orbital Size 1.0 (Thermofisher, Carlsbad, USA). During this process, microglia and OPCs that grew on the astrocyte layer detached gradually, while astrocytes adhered to the dish firmly.

For astrocyte purification, the remaining cells were digested with 0.05% Trypsin/EDTA (w/v) and plated into uncoated petri dishes, followed by incubating in a 5% CO_2_, 37 °C incubator for 30 min. The floating cells were removed and rinsed twice with 1× PBS. The remaining cells were cultured in D/F20S medium to confluency. Above purification procedure was performed two to three cycles to obtain high-purity astrocytes.

### Generation of lentiviral particles

For viral production, pCDH-Sox10 and pCDH-Sox10-GFP plasmids were obtained by inserting Sox10 ORF into pCDH vectors after the CMV promoter. Plasmids were prepared as previously described (Yang and Yang 2012). Lentiviral particles were generated by transfecting 293 T cells. Supernatants were collected after 48 h, filtered through an 0.45 μm polyvinylidene difluoride filter (Merck, Darmstadt, Germany) and concentrated overnight. The viral titer was determined as previously described (Jiang et al. 2003), and stored at − 80 °C.

### Induction of the transdifferentiation of astrocytes to oligodendrocytes in vitro

Purified astrocytes were placed into a 12-well plate at a density of 3 × 10^4^ cell/cm^2^, and infected with the Sox10 or Sox10-GFP virus on the next day. 6 h after the viral infection, the original medium was discarded and cells were washed twice with 1× PBS and replaced with fresh glial basal media (DMEM/F12 supplemented with 1× N2, 1× B27 and 1× P/S, all from Gibco, Carlsbad, USA) containing 10 ng/ml PDGFaa (PeproTech, Rocky Hill, USA). Medium was replaced every other day until d15.

### Preparation of O4^+^ iOPCs by immunopanning

Immunopanned Petri dishes were prepared as previously described (Grinspan et al. 2000; Neman and de Vellis 2012). For obtaining more O4^+^ iOPCs, we used 100 mm petri dishes for transdifferentiation culture and extended the transdifferentiation culture time to 21 days. Then cells were digested with 0.05% Trypsin/EDTA (w/v), and were subsequently seeded at 2.5 × 10^6^ per 100 mm uncoated dish and cultured for 30 min to enable astrocytes to attach to the dishes. After the incubation, the non-adherent cells were collected, and seeded into dishes coated with the O4 antibody and incubated for 30 min at room temperature. The adherent cells were then removed by treatment with 0.05% trypsin/EDTA (w/v) and replated onto the PDL/Laminin-coated dishes in glial basal media plus 10 ng/ml PDGFaa.

### iOPC differentiation

The directed differentiation of iOPCs derived from Sox10-GFP virus-induced transdifferentiation into mature oligodendrocytes was performed as previously described (Cheng et al. 2017). Briefly, iOPCs were cultured in PDL/Laminin-coated chamber slides (Millipore, Bedford, USA) with glial basal media supplemented with 30 ng/ml Triiodothyronine (T3) (Sigma-Aldrich, St. Louis, USA) for 3 days, then examined for MBP antigen expression by immunostaining.

### Dorsal root ganglion neuron (DRG) co-culture for myelination assays

iOPCs that are used to test myelination ability were induced with pCDH-Sox10 lentivirus that does not express GFP, so that the iOPCs obtained were all GFP negative. DRGs were isolated from E15 Sprague–Dawley rat embryos, the experimental procedures were carried out in accordance with Hangzhou Normal University IACUC guidelines. After trypsinization, DRGs were mechanically dissociated using a flame-polished Pasteur pipette, and the cell suspension was transferred into a 15 ml centrifuge tube and centrifuged at 200×*g* for 5 min at room temperature. After removing the supernatant, culture medium (DMEM-F12 supplemented with 10% FBS, 10 ng/ml nerve growth factor (NGF, PeproTech, Rocky Hill, USA), and 1× P/S) was added to resuspend the pellet, and the cell suspension was plated onto poly-l-lysine-coated dish. Culture medium was replaced on the following day and changed every 2 days thereafter. Contaminant non-neuronal supporting cells were eliminated by treatment with 4 μM of 5-fluoro-2-deoxy-uridine (FUDR) plus 4 μM of uridine (Sigma-Aldrich, St. Louis, USA) in a 5% CO_2_, 37 °C incubator for 3–5 days. DRGs were cultured for neurite outgrowth as described by Dincman et al. (2012). iOPCs/DRGs co-cultures were maintained for 8–12 days in glial basal media supplemented with 30 ng/ml T3, and fixed for immunostaining with anti-MBP and anti-neurofilament (axons) antibodies.

### qRT-PCR

For quantitative real-time reverse transcription (qRT) PCR, cells were lysed, and RNA was isolated using RNAiso Plus (9109, Takara) according to the manufacturer’s manual. 1 μg RNA were subjected to complementary DNA (cDNA) generation using the cDNA Synthesis kit (Takara, Kusatsu, Japan). Samples were then subjected to quantitative PCR analysis on a Bio-Rad CFX96 Real Time PCR System. The following primer pairs were used: 5′-TTTCTCCAACCTCCAGATCC-3′ and 5′-CCGCATCTCCACAGTCTTTA-3′ for GFAP; 5′-GCAGCCACCTATCTCCT CATC-3′ and 5′-CGAGTAGGGTAGGATAACTTCGC-3′ for Olig1;

5’-GGCGGTGGCTTCAAGTCAT-3’ and 5′-CATGGCGATGTTGAGGTCG-3′ for Olig2; 

5’-ACCAAAAGCAACGGAGAAGAG-3’ and 5′-GGCATTCCGAAACAGGTAACTC-3′ for Glast; 5′-GAAGCGCATGTCGAAAGAAGA-3′ and 

5’-GGCGGAGGCAGTCAATTCTC-3’for NFIA; 5′- AGTACCCGCATCTGCACAAC-3′ and 5′-ACGAAGGGTCTCTTCTCGCT-3′ for Sox9. Relative mRNA level was normalized against the house keeping gene GAPDH.

### Surgical procedures

All experimental procedures were carried out in accordance with institutional guidelines for the care and use of laboratory animals, and the protocol was approved by the Animal Ethics Committee of Hangzhou Normal University, China. After intraperitoneal injection of tamoxifen (75 mg/kg body weight) for 5 days, adult *hGFAP*^*Cre−ER*^*:Rosa26-tdTomato* transgenic mice were anesthetized with Nembutal (50 mg/kg, i.p.) and received a dorsal laminectomy at the tenth thoracic vertebral level (T10) to expose the spinal cord, and then a 75 kdyn contusive SCI using the IH-0400 Impactor (PSI, Fairfax Station, USA). At 2 days after injury, mice were randomly assigned to four groups, which received PBS, Sox10 virus, Sox10 virus + Gefitinib (EGFR inhibitor), Sox10 virus + EGF (PeproTech, Rocky Hill, USA), or Sox10 virus + EGF + Gefitinib (Sigma-Aldrich, St. Louis, USA), respectively. Animals were re-anesthetized as above, and the laminectomy site was re-exposed. Virus or vehicle injections were made at the center of the lesion at depth 0.7 mm. 2 μl of Sox10 lentivirus (1 × 10^8^ IU/μl) or vehicle was injected through a glass micropipette with an outer diameter of 50–70 μm at rate of 0.5 μl/min. And then, Alzet® Osmotic Pumps (ALZET, Cupertino, USA) were installed according to the manufacturer’s manual, and PBS, EGF (10 ng/μl), or EGF (10 ng/μl) plus Gefitinib (1 μM) were continuously delivered (0.11 μl/h) in the lesion site for 21 days. Analgesics and antibiotics were administered post-surgically and daily for the next 3 days to reduce pain and prevent dehydration and infection.

### Immunostaining

Immunochemical analysis was carried out as previously described (Yang et al. 2016). Anti-mouse O4 IgM (50%, v/v) were produced by hybridoma culture. Anti-mouse Olig2 (1:1000; Oasis Biofarm), anti-mouse GFAP (1:1000; Oasis Biofarm), and anti-rabbit neurofilament (NF-1) (1:1000) were purchased from Merck (Darmstadt, Germany). Anti-rat MBP (1:500), anti-rabbit EGFR (1:200) and anti-mouse CC1 (1:500) was obtained from Abcam (Boston, USA). Anti-mouse Nestin (1:1000) and the Alexa-488 or Alexa-594 conjugated secondary antibodies were purchased from Invitrogen (Frederick, USA). The nucleic acid dye 4′,6-diamidino-2-phenylindole (DAPI) was obtained from Roche (Basel, Switzerland). 

### Western immunoblotting

Western blotting was carried out as previously described (Liu et al. 2020). Primary antibodies were used as follows: anti-Olig2 (1:3000), anti-GFAP (1:1000), anti-Nestin (1:1000, MA1-110, Invitrogen), anti-Erk1/2 (1:5000, ab184699, Abcam), and anti-p-Erk1/2 (1:5000, ab76299, Abcam). Horseradish peroxidase (HRP)-conjugated secondary antibody (Promega) was used at 1:2500. Chemiluminescent signals were detected by autoradiography using the ECL System (Amersham, Piscataway, USA).

## Results

### EGF promotes the transdifferentiation of astrocytes into iOPCs

**Fig. 1:**
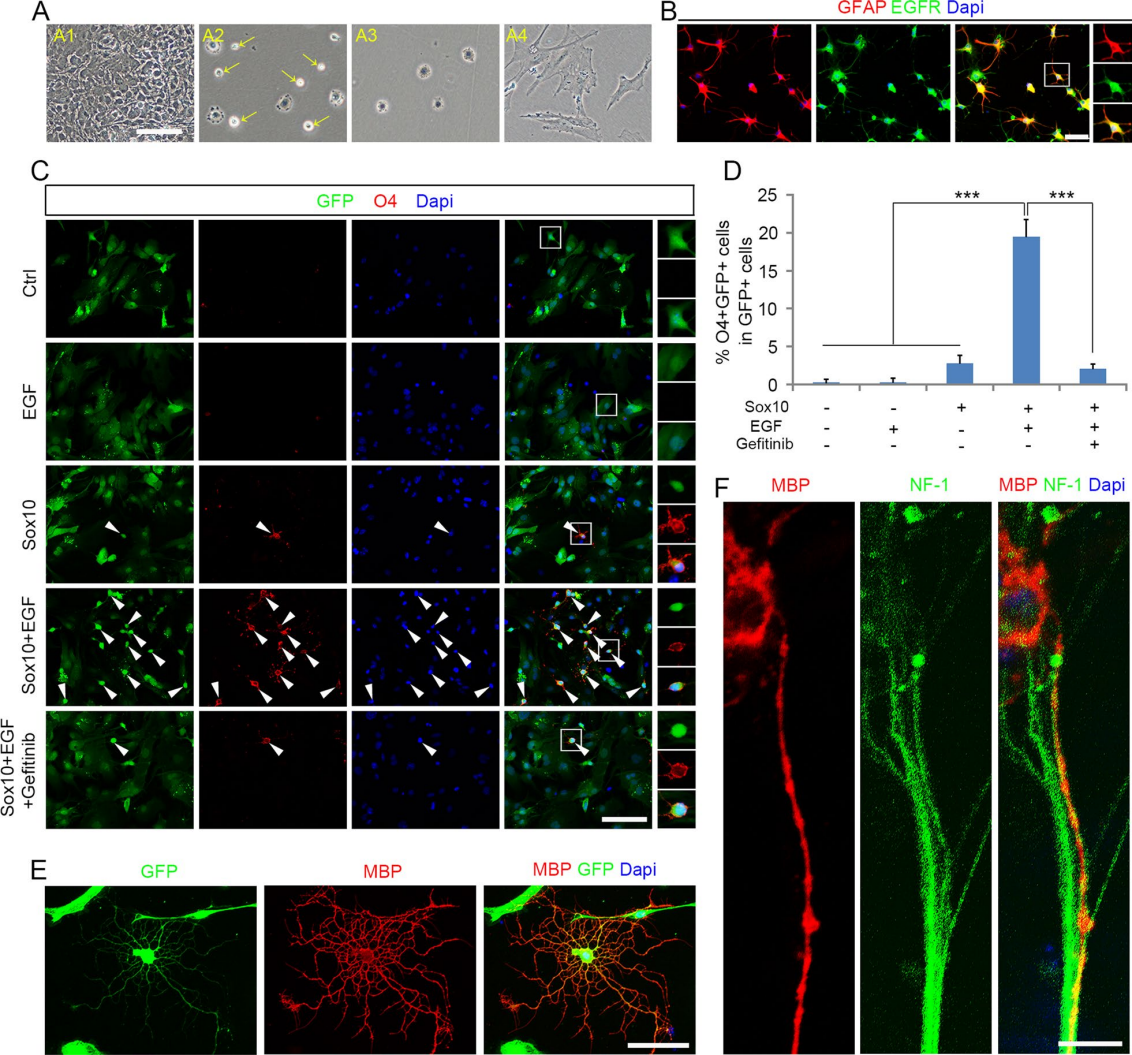
Sox10 and EGF act synergistically to transdifferentiate astrocytes into iOPCs. **A** Representative images of astrocyte purifying. (A1) Primary glial culture of mouse spinal cord tissue. (A2) The cells shown in A1 were passaged to uncoated dishes and cultured for 30 min, astrocytes quickly adhered to the surface of dishes, while other cells were still floating (yellow arrows). (A3) The floating cells were rinsed and astrocytes were retained. (A4) Astrocyte morphology 24 h after purification. **B** Purified cells were immunoreactive to GFAP and EGFR antibodies. **C** Representative images of the astrocyte transdifferentiation (15 dpi) in the five cultures. **D** Quantifications of experiments presented in **C**. **E** MBP^+^ mature OLs derived from iOPCs when cultured in the differentiation medium. **F** Confocal image of myelinated axon in iOPC/DRG co-culture. Purified iOPCs matured into MBP^+^ OLs in the co-culture, and aligned with axons marked by neurofilament (NF-1) staining. Statistical analyses are presented as Mean ± SD, n = 3. ***P < 0.001. Scale bars, **A**–**C** 50 μm; **E** 25 μm; **F** 10 μm

Astrocytes were obtained from mouse spinal cord dissociation culture by differential adherent properties, as astrocytes quickly adhered to the surface of uncoated culture dishes, while other cell types were less adherent and subsequently eluted out (Fig. 1A, B). Nearly all purified cells express GFAP, and they did not express TUJ1 (neuronal marker) and Sox10 (oligodendrocyte lineage markers) (Additional file 1: Fig. S1). Like reactive astrocytes in vivo (Liu and Neufeld 2007), these GFAP^+^ astrocytes are immune-positive for epidermal growth factor receptor (EGFR) (Fig. 1B), suggestive of their potential responsiveness to EGF. Astrocytes were infected with Sox10-GFP lentivirus, and cultured in a serum-free glia basal media plus PDGFaa. The expression of O4 antigen can be detected in OPCs and premyelinating oligodendrocytes (Yang et al. 2011), and therefore was used as a marker to examine the transdifferentiation of astrocytes into oligodendrocytes. After 15 days of culture, only a very few cells were transformed into O4^+^ oligodendrocytes (Fig. 1C, D), contrary to the earlier finding that overexpression of Sox10 in vivo induced astrocytes to transdifferentiate into oligodendrocytes (Khanghahi et al. 2018). Therefore, we suspected that the transdifferentiation may require additional factors that are present in the pathological microenvironment.

Since EGFR is highly expressed in astrocytes, EGF is an excellent candidate for the co-stimulating factor. Therefore, EGF was tested in the Sox10-GFP viral transfection cultures. Immunostaining results revealed that a fraction of the cells (19.5 ± 2.3%) started to express O4 antigen 15 days post infection (dpi) (Fig. 1C, D). EGF treatment alone in the absence of Sox10-GFP virus failed to induce O4^+^ oligodendrocyte (Fig. 1C, D), suggesting the lineage conversion requires the collaboration between Sox10 and EGF. Consistently, addition of EGFR inhibitor Gefitinib to the culture medium abolished the transdifferentiation effect of EGF and Sox10 treatment (Fig. 1C, D). The induced O4^+^ cells displayed typical morphology of oligodendrocyte precursor cells (OPCs) (Fig. 1C). Under T3 stimulation, they further differentiated into MBP^+^ mature oligodendrocytes (Fig. 1E) and formed myelin sheaths around the NF-1^+^ axons when co-cultured with DRG neurons (Fig. 1F). These results indicated that EGF promotes the Sox10-mediated astrocyte fate switch to iOPCs which are capable of further differentiating into myelinating OLs.

### EGF promotes the dedifferentiation of astrocytes into astrocyte precursor cells (APCs)

**Fig. 2:**
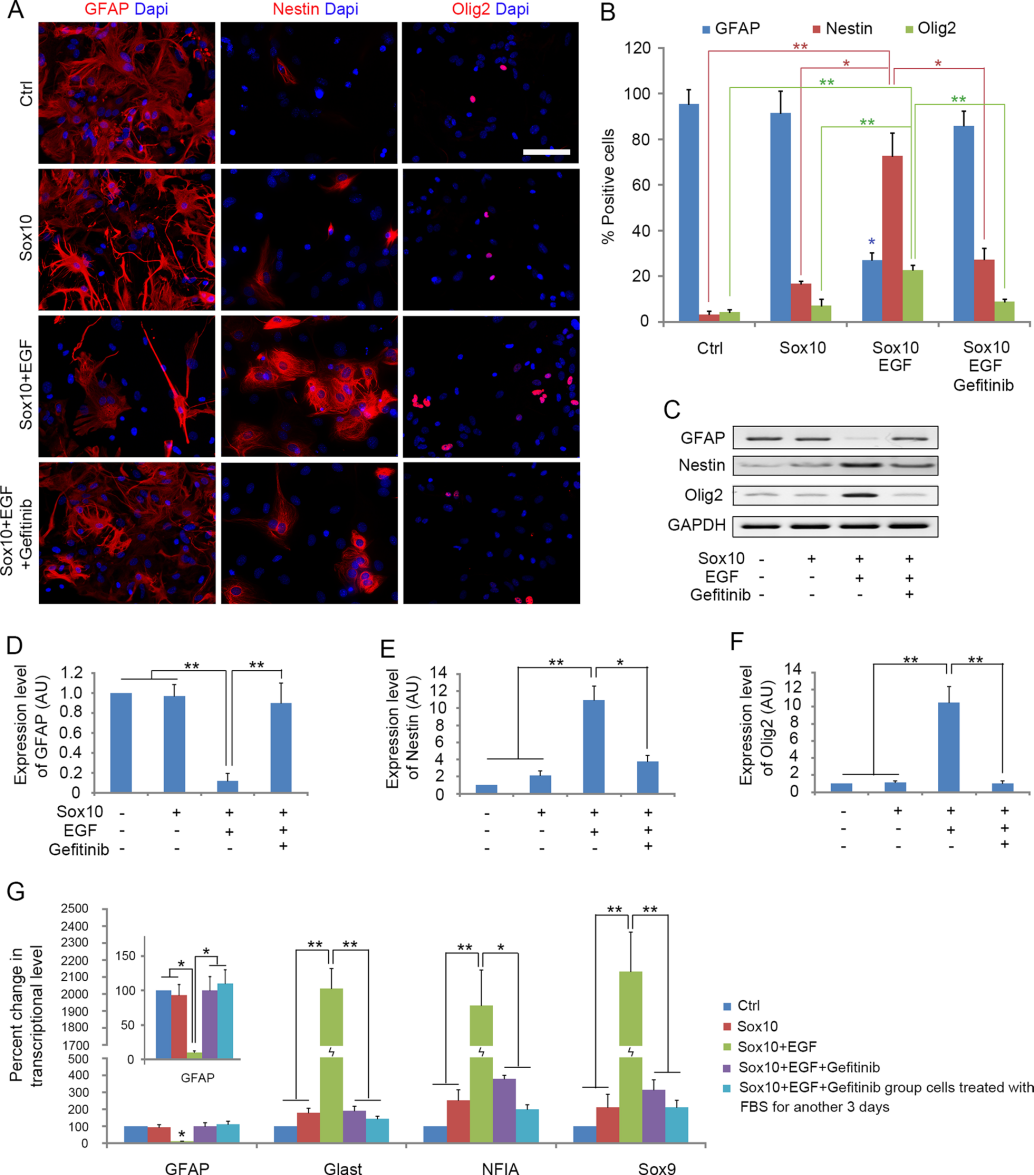
EGF promotes astrocyte dedifferentiation. **A** Immunostaining of cells with GFAP, Nestin and Olig2 antibodies 5 days after transdifferentiation. **B** Quantification of immune-positive cells in the four cultures. **C** Western blotting analysis of GFAP, Nestin and Olig2 on d5 during the transdifferentiation process. **D**–**F** Quantitative analysis of GFAP, Nestin and Olig2 proteins expression in the four cultures on d5, respectively, histograms express results in arbitrary units, taking Ctrl cells values as 100%. **G** Quantitative RT-PCR analysis of the expression level of GFAP, Glast, NFIA and Sox9 in the four groups on d5 during the transdifferentiation process and Sox10 + EGF + Gefitinib group cells treated with FBS for another 3 days. Statistical analyses are presented as Mean ± SD, n = 3. **P < 0.01, ***P < 0.001. Scale bars, 50 μm

We next examined whether Sox10-EGF-induced reprogramming of astrocytes to iOPCs is a direct transition between these two types of cells. When the transdifferentiation procedure progressed to the 5th day, the expression level of GFAP in infected astrocytes was significantly reduced (Fig. 2A, B). The majority of cells began to express Nestin (Fig. 2A, B) and several molecular markers for astrocyte precursor cells (APCs), including Glast, NFIA and Sox9 (Liu et al. 2004; Domowicz et al. 2011) (Fig. 2G), suggesting that at this stage, astrocytes started to dedifferentiate and reverse to the APC stage. Consistently, OPC-specific marker O4 antigen was not detected, and these cells could not differentiate into MBP^+^ OLs when cultured in differentiation medium (data not shown). Moreover, fetal bovine serum (FBS) treatment rapidly suppressed the expression of these precursor genes, but increased the expression of more mature astrocyte marker GFAP (Fig. 2G).

In the absence of EGF, overexpression of Sox10 only induced a small percentage of cells to express Nestin (Fig. 2A, B), and the expression levels of Glast, NFIA and Sox9 were also significantly lower than those in the Sox10 + EGF group (Fig. 2G). More importantly, a significant portion of cells (22.8 ± 1.9%) in the Sox10 + EGF group expressed Olig2 at d5 (Fig. 2A, B), while only few Olig2^+^ cells emerged in the Sox10 group at this time (Fig. 2B). In addition, EGFR inhibitor Gefitinib counteracted the effect of EGF in promoting dedifferentiation of astrocytes into APCs (Fig. 2A–G). Together, these results suggest that EGF promotes the dedifferentiation of astrocytes into APCs, the first step for enhancing astrocyte transdifferentiation into oligodendrocytes.

### The transition from APCs to O4^+^ iOPCs requires EGFR signaling

**Fig. 3:**
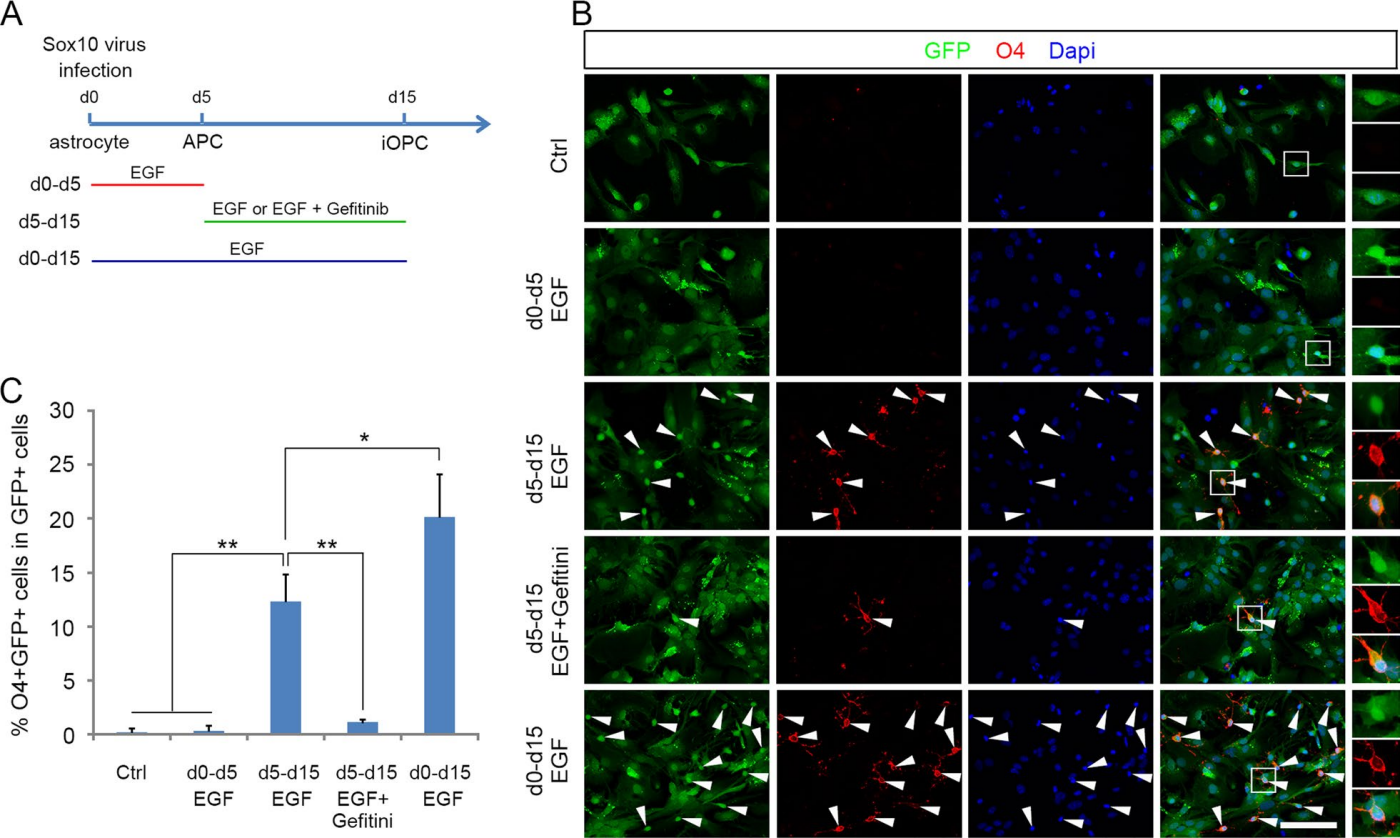
EGF promotes the switch of APCs to iOPCs. **A** The schematic of EGF and Gefitinib treatments at different time points during Sox10-induced reprogramming. **B** After infection with Sox10-GFP virus, the cells were cultured in five different culture conditions for 15 days, then immunostaining was performed with O4 antibody. **C** Quantification of the percentage of O4^+^ cells in five separate reprogramming cultures. Statistical analyses are presented as Mean ± SD, n = 3. *P < 0.05, **P < 0.01. Scale bars, 50 μm

To investigate the function of EGF in the process of transforming APCs to iOPCs, we treated the Sox10-GFP virus-infected astrocytes with EGF or its inhibitor for longer time periods as outlined in Fig. 3A. The results showed that treatment with EGF on d5–d15 induced 12.3 ± 2.4% of cells to express O4 antigen, but none in the control group (Fig. 3B, C). Gefitinib offsets the transdifferentiation effect of EGF, indicating that EGFR activation is necessary for the transformation process. To explore whether EGF treatment during the astrocyte dedifferentiation process contributes to the subsequent transformation into iOPCs, we compared the percentage of O4^+^ cells in the d5–d15 group with the d0–d15 group and the control group, and found that the percentage of O4^+^ cells in the d5–d15 group was markedly lower than that in the d0–d15 group. Consistent with the earlier observations with Olig2 expression, few O4^+^ cells were detected in the d0–d5 group (Fig. 3B, C). These results indicate that EGF treatment on d0–d5 significantly improved the efficiency of fate conversion. One plausible explanation for this finding is that EGF treatment yielded more Nestin^+^Olig2^+^ APCs, and the APCs obtained by EGF treatment were more readily to proceed into iOPCs.

The enhancement of astrocyte transdifferentiation to iOPCs by EGFR signaling was further validated by the inhibitory effect of Gefitinib (Fig. 3B, C). This finding is consistent with the phenomenon that EGFR signaling is required for the generation of pre-OPCs during in vivo development (Huang et al. 2020).

### EGF signaling promotes astrocyte transdifferentiation through the Erk cascade

**Fig. 4:**
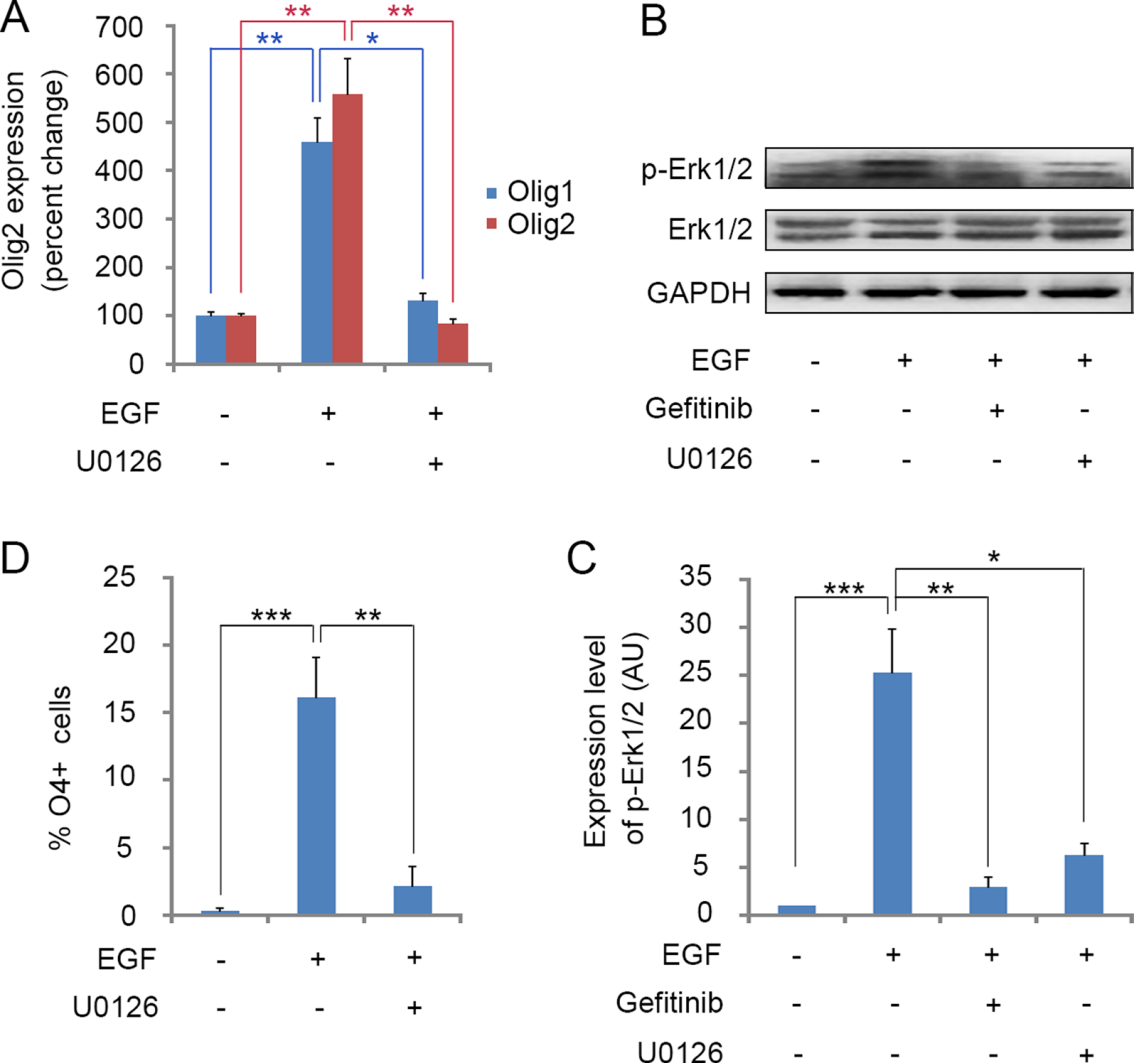
EGF induces the expression of oligodendrogenic genes through Erk pathway during Sox10-based reprogramming. **A** Gene expression of Olig1 and Olig2 after 36 h of EGF and EGF + U0126 treatment. **B** Western blotting analysis of p-Erk1/2 and Erk1/2 after 2 h of EGF, EGF + Gefitinib and EGF + U0126 treatment. **C** Quantitative analysis of p-Erk1/2 and Erk1/2 protein expression in cultures presented in **B**. Histograms express results in arbitrary units, using Ctrl cell values as 100%. **D** Quantification of the percentage of O4^+^ cells in reprogramming cultures treated with EGF or EGF + U0126. Statistical analyses are presented as Mean ± SD, n = 3. *P < 0.05, **P < 0.01, ***P < 0.001

Expression of regulatory genes for oligodendrogenesis in Sox10-GFP virus-infected astrocytes were measured by quantitative RT-PCR. Crucial pro-oligodendrogenic genes, such as Olig1 and Olig2 were significantly upregulated in EGF-treated astrocytes compared with control cells (Fig. 4A). It was found EGF treatment significantly increased the phosphorylation of Erk1/2 (Fig. 4B, C). Activation of Erk1/2 was blocked by both EGFR inhibitor Gefitinib and Erk inhibitor U0126 (Fig. 4B, C).

We next investigated whether Erk activity mediates EGF activation of downstream regulatory genes by adding U0126 compound to the EGF-treated astrocyte cultures and then measuring the expression of Olig1 and Olig2. U0126 treatment was found to produce a significant inhibition of EGF-induction of Olig gene expression (Fig. 4A). Meanwhile, pharmacological inhibition of Erk1/2 signaling in astrocytes also resulted in a significant reduction of O4^+^ iOPCs (Fig. 4D). These observations suggest that the Erk1/2 pathway (Plotnikov et al. 2011) is a major downstream mediator of EGF-dependent activation of astrocytic transdifferentiation process.

### EGF infusion promotes the transdifferentiation in vivo

**Fig. 5:**
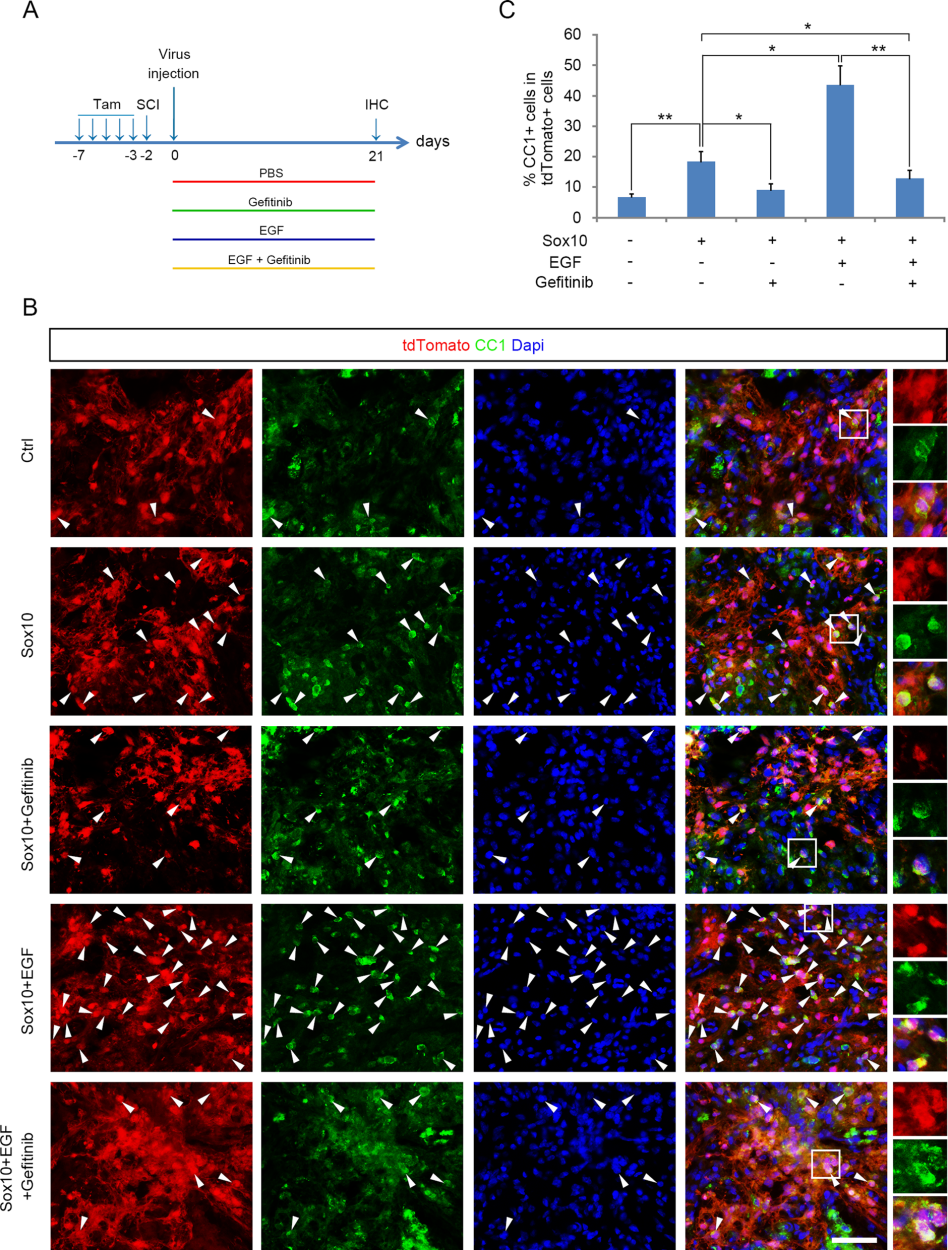
EGF infusion promotes the transdifferentiation of astrocytes to oligodendrocytes in vivo. **A** Experimental design for analyzing the effect of EGF signaling during Sox10 virus-induced reprogramming in vivo. **B** Representative pictures of *hGFAPCre-ER:Rosa26-tdTomato* transgenic mice infused with PBS, Gefitinib, EGF or EGF + Gefitinib during Sox10-based reprogramming and immunostained for CC1. Scale bars, 50 μm. **C** Quantification of the percentage of CC1^+^ oligodendrocytes. Statistical analyses are presented as Mean ± SD, n = 3. *P < 0.05, **P < 0.01

To test whether EGF enhances astrocyte transdifferentiation to oligodendrocyte in vivo, we labelled astrocytes in *hGFAP*^*Cre−ER*^*:Rosa26-tdTomato* double transgenic mice by intraperitoneal injection of tamoxifen for 5 days (Fig. 5A). Sox10 virus was then injected to lesion site 2 days after spinal cord contusion, followed by installation of Alzet® Osmotic Pumps (ALZET) for continuous delivery of PBS, Gefitinib, EGF or EGF + Gefitinib in the injury site (Fig. 5A). Immunohistochemical analysis was performed on spinal cord tissue and the percentage of tdTomato^+^ astrocytes that differentiated into oligodendrocyte lineage cells was quantified for five groups of mice. Sox10 overexpression significantly increased the percentage of tdTomato^+^CC1^+^ induced oligodendrocytes compared to the control tissue (Fig. 5B, C). However, it was attenuated by EGFR inhibitor Gefitinib (Fig. 5B, C), suggesting that EGF presented in the pathological milieu participated in the astrocyte-to-oligodendrocyte reprogramming. In further support, continuous delivery of EGF into injury area during transdifferentiating significantly increased the percentage of tdTomato^+^CC1^+^ induced oligodendrocytes (Fig. 5B, C) compared to the control tissue that overexpressed Sox10 only. EGFR inhibitor Gefitinib counteracted the synergistic effect of EGF during transdifferentiating, and reduced the number of CC1^+^ cells to a level similar to that of the Gefitinib group (Fig. 5C). Thus, EGF treatment can significantly elevate the efficiency of astrocyte transdifferentiation to oligodendrocytes in vivo as well.

## Discussion

Earlier studies demonstrated that following CNS injury, a small number of endogenous oligodendrocytes and surrounding invading Schwann cells can participate in remyelination (Gledhill et al. 1973; Itoyama et al. 1983). However, later studies in humans and rodents revealed that nerve impulse conduction is still blocked, indicating that spontaneous remyelination is very limited (Alexeeva et al. 1998; Nashmi and Fehlings 2001). The therapeutic strategy of converting activated astrocytes to induced OLs has several potential advantages. First, astrocytes are widely distributed throughout the CNS and could provide a rich source for oligodendrocyte reprogramming. Secondly, transdifferentiation of activated astrocytes into oligodendrocytes will help reverse glial scar tissue, thereby promoting axon regeneration and remyelination. Finally, compared with stem cell transplantation, this strategy of using activated astrocytes for in situ tissue repair evokes fewer immunological and ethical issues (Khanghahi et al. 2018). Exploring the molecular mechanisms underlying the conversion of astrocytes into oligodendrocytes will no doubt help to achieve more effective regeneration and repair. In this study, we discovered that EGF signaling greatly promotes this fate transition process both in vitro and in vivo.

Emerging evidence indicates that EGFR signaling plays an important role in oligodendrogenesis (Aguirre et al. 2007; Chong et al. 2008; Gonzalez-Perez et al. 2009; Hu et al. 2010; Huang et al. 2020; Fu et al. 2021; Li et al. 2021). While EGFR knock-out appeared to reduce the density of OPCs and mature oligodendrocytes in corpus callosum (Aguirre et al. 2007), enhancing EGFR signaling by intraventricular infusion of EGF induced type B cells to differentiate into oligodendrocytes (Gonzalez-Perez et al. 2009). It was also shown that the administration of intranasal heparin-binding EGF immediately after brain injury decreases oligodendrocyte death and enhances generation of new oligodendrocytes (Scafidi et al. 2014). In line with these findings, we discovered that EGF predisposes glial progenitor cells to develop into OPCs in vitro (Yang et al. 2017). More recently, ‘‘EGFR^+^ pre-OPCs’’ were identified in developing human cerebral cortex, which originate from outer radial glial cells, and EGF treatment resulted in a dose-dependent increase in the numbers of Olig2^+^Nkx2.2^+^ OPCs (Huang et al. 2020). The responsiveness of pre-OPCs to EGF suggests a tight regulation of OPC production by EGF secreted in the cortical cell niche (Huang et al. 2020). Together, these studies suggested that EGFR signaling plays an active role in oligodendrogenesis.

**Fig. 6:**
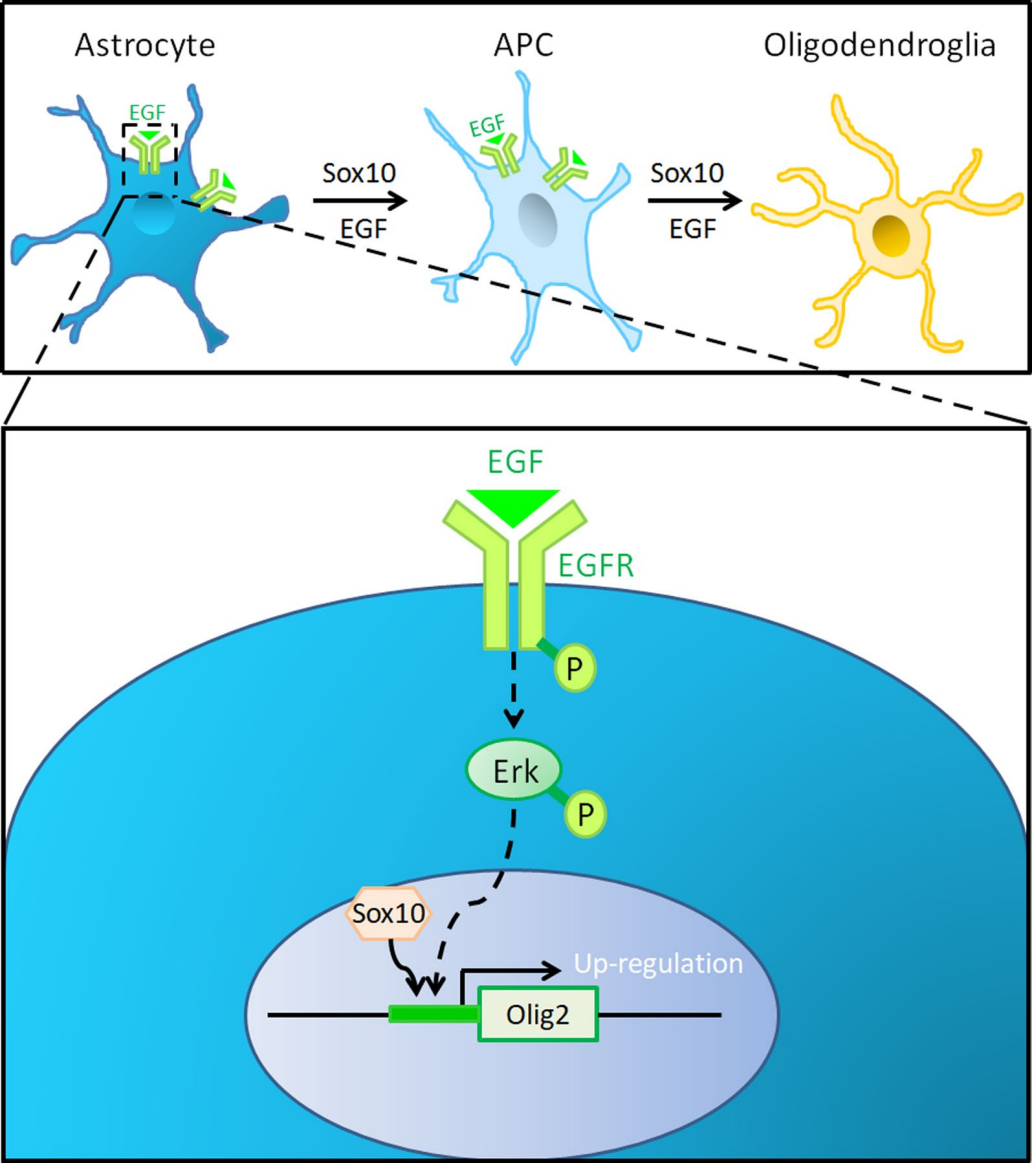
Proposed model of synergistic induction of transdifferentiation from astrocytes to oligodendrocytes by EGF and Sox10. EGF binds to EGFR to activate the downstream Erk pathway, thereby up-regulating the expression of oligodendrogenic genes, meanwhile, forced expression of Sox10 in astrocytes also promotes Olig2 expression and facilitates astrocyte reprogramming to iOPCs via APCs

Our results showed that EGF promoted Sox10-induced astrocyte transdifferentiation both in vitro and in vivo, which further strengthened our understanding of the function of EGF-EGFR signaling on oligodendrogenesis. Our detailed analyses revealed that EGF signaling promotes the dedifferentiation of astrocytes into APCs first, followed by the progression of APCs to OPCs in collaboration with the OPC-specific transcription factor Sox10 (Fig. 6A). Among the Nestin^+^ cells derived from the dedifferentiation of astrocytes, some might even dedifferentiate to the stage of basal multipotent intermediate progenitor cells (Huang et al. 2021). While this study demonstrated that EGF can induce the expression of oligodendrogenic genes Olig1 and Olig2, we previously demonstrated that Sox10 activity can stimulate Olig2 expression in neural progenitor cells to enhance oligodendrogenesis (Liu et al. 2007). Thus, it is possible that forced expression of Sox10 in astrocytes can promote Olig2 expression and facilitates astrocyte reprogramming to iOPCs (Fig. 6B). In spinal cord injury tissues, EGF expression was significantly increased (Ahn et al. 2006), which may explain why Sox10 overexpression in the injury environment was sufficient to induce astrocytes to transdifferentiate into oligodendrocytes, but failed to do so in vitro. In support of this concept, the transdifferentiation of astrocytes into oligodendrocytes in injury spinal tissues can be blocked by EGFR inhibitor Gefitinib. Recent studies have shown that chemical molecules such as Neuregulin-1, trichostatin A, 5-azacytidine and Ethyl Pyruvate can also induce the transdifferentiation of astrocytes into oligodendrocyte lineage (Zare et al. 2018; Ding et al. 2021; He et al. 2021). It is possible that EGF may cooperate with these molecules to promote remyelination, and EGF-containing cocktail could be developed as a molecular tool to promote CNS injury repair.

In addition to EGF, EGFR ligands also include heparin-binding EGF-like growth factor, β-cellulin, transforming growth factor alpha, amphiregulin, epiregulin, epigen, neuroregulin and Chitinase 3-like-3 (Galvez-Contreras et al. 2013; Starossom et al. 2019). Theoretically, these molecules are also potential promoters for the astrocyte transdifferentiation into oligodendrocytes. Exploring the expression and function of these molecules in the pathological microenvironment will further define the molecular natures of the extracellular signals that promote the in vivo transdifferentiation process in injured CNS tissues.

The major signaling pathways initiated by EGFR activation include: extracellular signal-regulated kinase 1 and 2 (Erk1/2) cascade, P38MAPK cascade, c-Jun N-terminal kinase cascade, signal transducers and activators of transcription cascade and phospholipase C-gamma cascade (Galvez-Contreras et al. 2013). We demonstrated that EGF stimulation in astrocytes also significantly increased the phosphorylation level of Erk1/2. Pharmacological inhibition analysis showed that the upregulation of Erk1/2 phosphorylation is necessary for upregulation of pro-oligodendrogenic genes Olig1 and Olig2 (Lu et al. 2002; Takebayashi et al. 2002; Zhou and Anderson 2002), and subsequent transdifferentiation of astrocytes to oligodendrocytes. However, we can not rule out the possibility that EGF also influences cell fate transdifferentiation through other downstream signaling cascades, such as those involved in cellular survival and proliferation (Plotnikov et al. 2011).

## Conclusion

In summary, we found that EGF synergizes with Sox10 to transdifferentiate astrocytes to oligodendrocytes both in vitro and in vivo, and it plays an important role in both the dedifferentiation of astrocytes to APCs and the transformation of APCs to oligodendrocytes. This effect was accompanied by the up-regulation of oligodendrogenic genes and mediated by the activation of MAPK pathway in an Erk1/2-dependent fashion. Our results implicate EGF-EGFR-Erk1/2 as a key intrinsic pathway influencing the astrocyte-to-oligodendrocyte fate change.

## Supplementary Information

**Additional file 1: Figure S1.** Most of the purified cells were GFAP immunopositive astrocytes.

## Data Availability

Not applicable.

## Abbreviations

iOPC: Induced oligodendrocyte precursor cell
Erk1/2: Extracellular signal-regulated kinase 1 and 2
APC: Astrocyte precursor cell
EGF: Epidermal growth factor
CNS: Central nervous system
OL: Oligodendrocyte
MAPK: Mitogen-activated protein kinase
OPC: Oligodendrocyte precursor cell
DRG: Dorsal root ganglion neuron
T3: Triiodothyronine
EGFR: Epidermal growth factor receptor
FBS: Fetal bovine serum
iOL: Induced oligodendrocyte
