# Supplementary material for: EGF signaling promotes the lineage conversion of astrocytes into oligodendrocytes
Source: Mol Med. 2022 May 4;28:50. doi: 10.1186/s10020-022-00478-5 (PMC9066914; doi:10.1186/s10020-022-00478-5)
Supplement: Supplementary file 1 — Additional file 1: Figure S1. Most of the purified cells were GFAP immunopositive astrocytes. [file 10020_2022_478_MOESM1_ESM.docx]

**Additional Material**


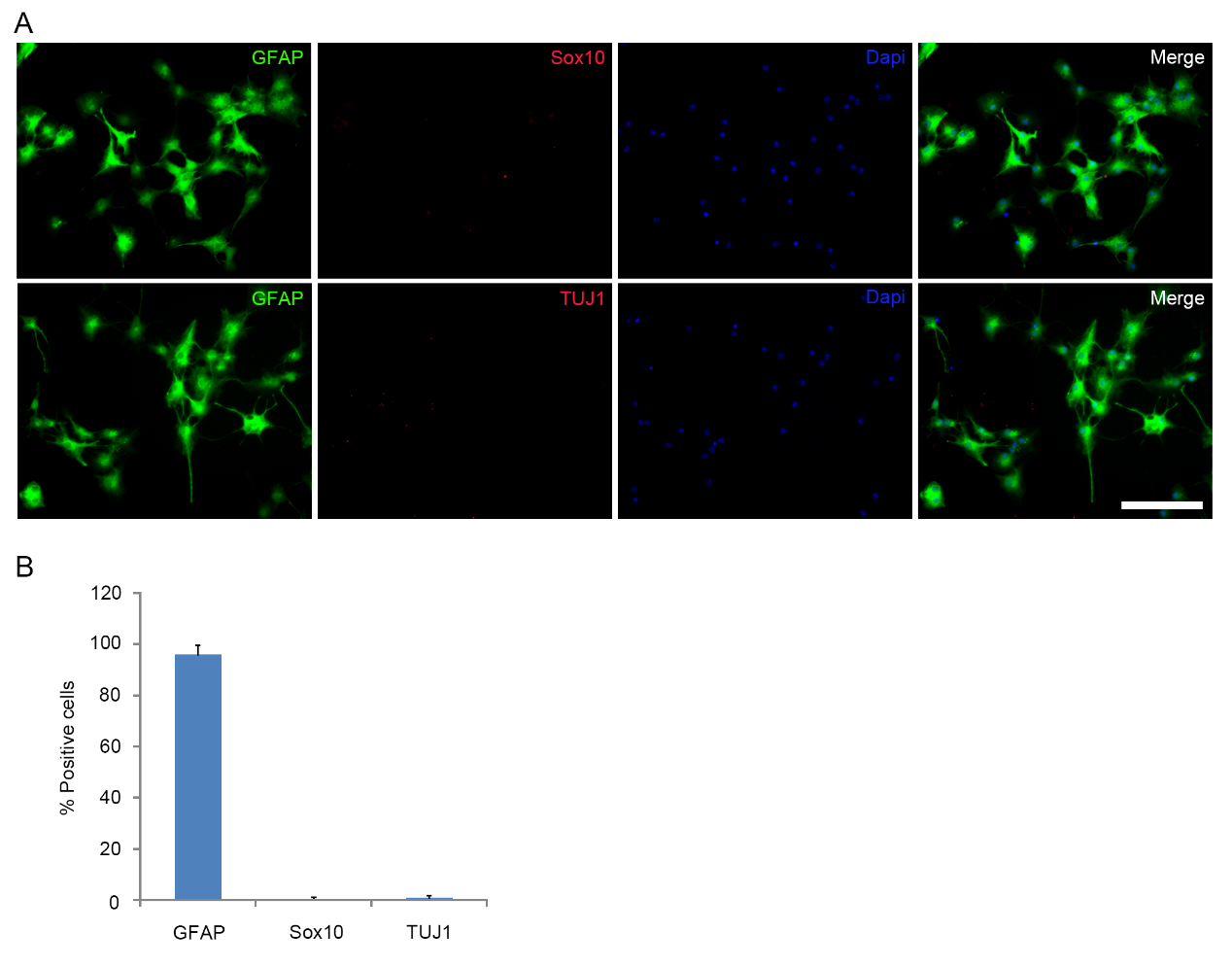


**Fig. S1** Most of the purified cells were GFAP immunopositive astrocytes.

A. Representative images of immunofluorescence staining of purified cells.

B. Quantifications of experiments presented in A.

Statistical analyses are presented as Mean ± SD, n = 3. Scale bars, 100 μm.
